# Adolescent physical activity during school days: a comparative study before and after COVID-19 pandemic restrictions

**DOI:** 10.3389/fpubh.2025.1488153

**Published:** 2025-03-14

**Authors:** Karel Frömel, Gregory Welk, Dorota Groffik, Lukáš Jakubec, Jan Dygrýn, Josef Mitáš

**Affiliations:** ^1^Faculty of Physical Culture, Palacký University Olomouc, Olomouc, Czechia; ^2^Faculty of Physical Education, The Jerzy Kukuczka Academy of Physical Education in Katowice, Katowice, Poland; ^3^Department of Kinesiology, Iowa State University, Ames, IA, United States

**Keywords:** physical activity recommendation, active transport, school day, education policy, COVID-19

## Abstract

**Background:**

Physical activity (PA) during the school day is crucial for the health and well-being of adolescents. This study examines the impact of the COVID-19 pandemic on youth PA patterns to better understand these changes and to provide guidelines for school programming.

**Methods:**

Differences in PA within specific segments of the school day were examined before and after the pandemic using the Youth Activity Profile questionnaire. Participants included 956 boys and 1,317 girls from 21 high schools. The study involved 12 Czech and 9 Polish high schools before the pandemic and 9 Czech and 8 Polish high schools after the pandemic.

**Results:**

Both Czech and Polish boys and girls exhibited significantly less transportation PA to and from school and reduced PA during the school day after the pandemic compared to before. Additionally, Czech and Polish boys were significantly less physically active during school breaks, and Czech boys and girls had notably less PA during physical education lessons. The pandemic disrupted the habit of regular PA on school days, particularly evident in the decline of PA to and from school.

**Conclusion:**

The study confirms a significant difference in PA of Czech and Polish adolescents in various segments of the school day after students return to school following pandemic restrictions. Promoting achievement of the recommendations in the segments of the school day and in comprehensive school PA programs should be an important part of school health and education policy and public health promotion for adolescents.

## 1 Introduction

Worldwide, 81% of adolescents aged 11–17 years are insufficiently physically active (1). Schools therefore play an important role in ensuring that youth achieve recommended levels of physical activity (PA) and build habits for lifelong PA (2, 3). Active lifestyle in schools, along family and community involvements, is crucial for supporting healthy lifestyle in adolescents (4). Schools are most effectively positioned to influence the vast majority of children and youth during this critical period of their development (5). Thus, schools have an opportunity and obligation to positively influence student’s psychological (6), physical (7) and social development during this “sensitive” developmental period (8). The school curriculum is the fundamental pillar for creating good work habits (9), realizing one’s own identity in the educational process (10) and realizing the importance of one’s own responsibility in the approach to a healthy lifestyle (11). However, it is also important to recognize the importance of school PA in compensating the educational load (12). Schools that promote PA across the day provide a better learning environment while also providing the opportunities youth need to meet public health guidelines. To ensure long-term habits are formed, schools also need to help youth to build cognitive and behavioral skills needed to PA (13). The benefits of regular PA for well-being are well documented (14) and school PA helps to ensure that adolescents recognize the feelings of satisfaction from PA and also transfer them to extracurricular PA (15, 16).

A critical need in youth PA promotion is to better understand the patterns of PA across a week and the relative contribution of PA that comes from segments of the school day as well as from home activities. The distribution of PA in individual school days of the week, together with weekend days, must be considered when creating comprehensive school PA programs and when creating weekly curriculum programs for physical education (PE). For example, Czech and Polish adolescents indicate most PA on Fridays (17, 18) and the least amount of PA on weekend days (19). This information can aid in planning PE and school programming.

School PA programming should provide opportunities for PA across the school day, but the most important contributor is PE (20). PE lessons help in building the motor skills for coping with new PAs in the future and creating habits for safe PA performance (21). It is still unclear, whether PE lessons are able to respond to accelerating changes in the lifestyle of adolescents and to respond to significant societal changes as well (i.e., the non-participation of adolescents in PE lessons). In an overview of 67 countries, Martins et al. (22) demonstrated that 18.2% of adolescents do not participate in PE lessons. In addition, we have evidence of decreasing level of assessment of PE lessons by Czech and Polish adolescents (23), as well as not meeting the recommendation of spending 50% of time in moderate to vigorous PA in PE lessons. Hollis et al. (24) reported that 35.9% of high school students achieved the moderate to vigorous PA level of intensity during PE lessons and Frömel et al. (25) reported only 25.2%. The compensatory role of breaks is unquestionable, but even with a total duration of 60 min they cannot replace PE lessons (26).

It is positive that there is global agreement on daily PA recommendations of at least 60 min of moderate to vigorous PA per day, supplemented by vigorous-intensity aerobic activities, as well as those that strengthen muscles and bones (27). Most PA recommendations focus on daily or weekly PA recommendations or PA related to school, especially active transport to and from school (14). The most well-known recommendations are trips to school of 1 mile and trips made by bicycling to school of 2 miles (28). A more comprehensive draft recommendation on PA in segments of the school day is based on Central European settings and has been applied in recent years (25).

The long-term global decline in adolescent PA (29) clearly worsened during the COVID-19 pandemic (hereafter referred to as the pandemic) (30–33). The pandemic demonstrated important deficiencies in the preparation of students for individual and home PA, for compensating the distance learning through PA, and for maintaining fitness levels when organized PA was interrupted (34). Schools were also not ready to provide PA with attractive online PE in distance education (32).

To address these issues in the future, it is important to better understand how the pandemic shifted overall PA patterns in youth and how this shifted when returning to the habitual curriculum after the pandemic.

Verification of research findings in different educational systems could support the strength of the evidence. PE is compulsory in both countries, but in Poland it is usually 3 h of PE per week, while in the Czech Republic it is 2 h per week. A higher number of weekly PE hours in Poland is positively associated with higher vigorous PA in adolescents (35). Similar is the excusing from PE for medical reasons, and the undesirable excuse for non-participation in PE. PE curricula are very similar, but in Poland there is a greater focus on performance sport in schools, which is related to the single-discipline professional focus of PE teachers. In the Czech Republic, teachers are mostly endorsed to teach PE and another subject.

Thus, the aim of the study is to determine the differences in the PA structure of Czech and Polish boys and girls in school days before and after the pandemic (after the end of pandemic restrictions in schools). The study capitalized on the time segmentation capabilities of the Youth Activity Profile (YAP) questionnaire to quantify differences and to propose a guideline for feedback information to schools.

## 2 Materials and methods

### 2.1 Study design and participation

We carried out the research in Czech and Polish high schools in two-year stages. In the years 2019–2020 (before the pandemic restrictions) and in the years 2021–2022 (after the end of nationwide restrictions and the gradual return of schools to the standard curriculum). Research before and after the pandemic was conducted in two seasons: autumn (September–November) and spring (March–May). Before the pandemic, we contacted high schools in the Czech Republic and Poland that cooperated regularly with the universities (12 Czech and 9 Polish schools agreed to participate). In the second stage, after the pandemic, we approached schools that had returned back to full-time education, as close as possible to a standard curriculum. These were different high schools than before the pandemic, but they were schools of the same type, size and regional distribution. (9 Czech and 8 Polish schools). Infrastructure in terms of school equipment was similar in both Czech and Polish schools.

Data were collected from 1,385 youth (aged 15–18) before the pandemic (833 girls and 552 boys) and from 888 youths after the pandemic (484 girls and 404 boys). Only 5% of boys and 5% of girls were overweight or obese (< 25 kg/m^2^). The descriptive statistics of the samples from the two countries are provided in Table 1. This is a stratified and quota-based set of schools and classes/groups of pupils selected for purpose. We selected groups of students at the schools on the date set by the school management, but in such a way that they had an information technology lesson in the computer lab included in the regular curriculum and there was no extreme weather in the week under consideration. All participating students and their parents signed informed consent. At the schools before the pandemic, more than 90% of the students surveyed agreed with the research, and after the pandemic, 10–15% of the students at two Czech and three Polish schools disagreed.

**Table 1:** Sample characteristics.

Characteristics	Pandemic	*n*	Age (years)		Weight (kg)		Height (cm)		BMI (kg·m^−2^)		PEL (n/week)		TL (n/week)	
			M	SD	M	SD	M	SD	M	SD	M	SD	M	SD
Boys CZ	Before	266	16.2	1.8	67.7	13.5	175.7	9.5	21.8	3.3	1.8	0.8	1.7	2.1
	After	258	16.3	1.1	67.8	11.4	178.1	8.0	21.3	3.1	1.3	0.7	1.7	2.1
Boys PL	Before	286	15.2	1.7	63.5	14.4	171.6	11.2	21.3	3.3	3.2	0.9	2.2	2.1
	After	146	15.5	1.0	65.4	12.2	175.8	8.1	21.1	3.1	2.3	0.8	1.8	2.0
Girls CZ	Before	500	16.6	1.7	58.1	8.6	166.3	6.6	21.0	2.8	1.7	0.7	1.1	1.5
	After	269	16.0	1.3	58.5	9.7	167.4	7.1	20.8	2.9	1.2	0.5	1.3	1.7
Girls PL	Before	333	15.2	1.7	54.8	10.2	163.0	6.4	20.6	3.2	2.9	0.9	1.7	1.9
	After	215	15.9	1.1	55.9	8.7	165.6	5.8	20.3	2.8	2.2	0.8	1.1	1.5

M, mean; SD, standard deviation; BMI, body mass index; CZ, Czech Republic; PL, Poland; PEL, physical education lessons; TL, after-school sport training lessons or organized PA with an officially designated leader.

The entire research was performed by the same research teams in both countries, always accompanied by a responsible administrator designated by the school management. School administrators were informed of the process of data anonymization (using codes) in the web application “International Database for Research and Educational Support” (Indares)^1^ and the processing of average group results for the needs of the schools involved.

### 2.2 Measure

The established Youth Activity Profile (YAP) questionnaire (36, 37) was used to assess the level of school-based PA of adolescents. The Czech and Polish versions were translated in accordance with the requirements of the EORTC Quality of Life Group (38). As part of the standardization of the Czech version of the questionnaire, we conducted monitoring of PA with accelerometers (ActiGraph, GT9X LINK and wGT3X+, ActiGraph Corp., Pensacola, FL, USA), and reported validity coefficient in the range of *r_s_* = 0.40–0.49 (39). On school days, a 10% equivalence zone of agreement was determined by the equivalence test. The final versions of the questionnaires are available in electronic form on the Indares web application.

The questions of the YAP are designed based on a five-point Likert scale. Questions about PE lessons, breaks and lunch breaks also include the option to answer that this event did not appear in the school schedule. The questionnaire includes six questions on the characteristics of the respondent in terms of attitudes to PA and PE. The basic questions are: “I enjoy doing physical activity” and “I enjoy physical education.” Also, two questions about transport PA: travel to school and from school – 0 days (never), 1 day, 2 days, 3 days, 4–5 days (most every day). Three questions about school PA: Activity during PE lessons –” *I did not have PE, almost none of the time, a little bit of the time, a moderate amount of time, a lot of the time, almost all of the time*“. Activity during breaks/study hall and activity during lunch breaks –” *I did not have breaks, almost none of the time, a little bit of the time, a moderate amount of time, a lot of the time, almost all of the time*“. And also, three questions about PA outside of school at least 10 min: Activity before school, activity after school and activity on evenings – 0 days, 1 day, 2 days, 3 days, 4 to 5 days. Other questions of the questionnaire are focused on PA on Saturday and Sunday and on sedentary habits.

This study focused only on the analysis of data representing school PA and school-related PA. The subjectively reported height and weight of the respondents was checked by the teacher but was only used to characterize the population. We evaluate individual PA levels in segments of the school day, as well as aggregated school PA (PE lessons, breaks and lunch breaks), aggregated outside school PA (to and from school, before and after school) and aggregated PA in the school day (before and after school, to and from school, school PA), always as a sum of points according to the Likert scale. For PA in the segments of the school day, based on the results of the research, we set target recommendations for PA in the individual segments of the school day. Emphasis was placed on interpreting raw YAP point scores instead of converted time estimates since the focus was on the overall changes and not for surveillance purposes.

### 2.3 Data analysis

For statistical analyses the software Statistica version 14.0.0.15 (StatSoft, Prague, Czech Republic) was used. We used basic descriptive statistics to characterize the file, Kolmogorov–Smirnov and Lilliefors tests to assess normality, and the Mann–Whitney U test to determine differences in PA in the segments of the school day before and during the pandemic. Furthermore, the Kruskal-Wallis ANOVA test was used for the summary assessment of school PA, outside school and total PA on school days. Differences in achieving goals in PA in segments of the school day were assessed by cross-tabulation. The *ŋ^2^* and *r* effect size coefficients were evaluated as follows: 0.01 ≤ *η^2^* < 0.06 (0.1 ≤ *r* < 0.3) small effect size, 0.06 ≤ *η^2^* < 0.14 (0.3 ≤ *r* < 0.5) medium effect size, *η^2^* ≥ 0.14 (*r* ≥ 0.5) large effect size. The level of statistical significance was established at *p* < 0.05.

## 3 Results

### 3.1 Differences in PA before and after the pandemic in segments of school days

After the pandemic, we found a significantly lower number of days with transport PA to and from school for both boys and girls in both countries compared to the period before the pandemic (Table 2). The highest number of days with transport PA before pandemic demonstrated Polish boys to and from school (3 times a week on average). After the pandemic Polish girls showed the greatest transport PA (to school on average 2.5 times and from school 2.7 times per week). Czech and Polish boys and Czech girls also showed significantly less use of time for PA during breaks after the pandemic. It is noteworthy that we found less use of time for PA in PE lessons after the pandemic only in Czech boys and girls. We also found less PA during lunch breaks and after school in Polish boys after the pandemic.

**Table 2:** Assessment of PA levels on school days in Czech and Polish boys and girls before and after pandemic.

Physical activity levels	Czech boys					Polish boys				
	Before M(SD)	After M(SD)	U	*p*	*η^2^*	Before M(SD)	After M(SD)	*U*	*p*	*η^2^*
School days										
Before school	0.8(1.4)	0.8(1.3)	0.17	0.866	< 0.001	1.2(1.5)	1.0(1.4)	1.45	0.148	0.007
To school	2.6(1.8)	2.2(1.8)	2.08	0.038	0.016	3.0(1.6)	2.4(1.8)	3.18	0.001	0.035
From school	2.9(1.6)	2.4(1.8)	3.55	<0.001	0.047	3.0(1.6)	2.5(1.7)	3.36	0.001	0.039
After school	2.4(1.4)	2.4(1.4)	0.16	0.870	< 0.001	2.3(1.4)	2.0(1.5)	2.46	0.014	0.021
Evenings	1.6(1.4)	1.7(1.5)	0.44	0.659	0.001	2.1(1.5)	1.9(1.5)	1.23	0.220	0.005
Time (moderate/average use of the time for PA in PE lessons or breaks)										
During PE lessons	4.0(1.0)	3.6(1.0)	3.94	<0.001	0.058	4.1(1.0)	4.1(1.0)	0.50	0.620	0.001
During breaks	2.2(1.3)	1.9(1.1)	2.75	0.006	0.028	2.6(1.4)	2.2(1.2)	2.76	0.006	0.027
During lunch breaks	2.2(1.2)	2.3(1.2)	1.11	0.268	0.005	2.6(1.3)	2.2(1.2)	2.53	0.011	0.022

	Czech girls					Polish girls				
School days										
Before school	0.8(1.4)	0.7(1.3)	0.71	0.478	0.001	0.9(1.3)	0.7(1.3)	2.23	0.026	0.015
To school	2.7(1.8)	2.1(1.8)	4.67	0.000	0.044	2.8(1.6)	2.5(1.8)	2.15	0.032	0.014
From school	2.9(1.7)	2.3(1.8)	5.11	0.000	0.052	2.9(1.6)	2.7(1.7)	2.07	0.039	0.013
After school	2.2(1.4)	2.2(1.4)	0.28	0.782	< 0.001	1.9(1.5)	1.8(1.4)	1.13	0.260	0.004
Evenings	1.4(1.3)	1.5(1.4)	1.01	0.312	0.002	1.8(1.4)	1.6(1.5)	1.66	0.097	0.003
Time (moderate/average use of the time for PA in PE lessons or breaks)										
During PE lessons	3.6(1.1)	3.3(1.1)	3.40	0.001	0.023	3.7(1.2)	3.7(1.0)	0.08	0.938	< 0.001
During breaks	1.8(0.9)	1.7(0.9)	1.97	0.049	0.008	2.0(1.2)	1.9(1.1)	0.83	0.405	0.002
During lunch breaks	2.1(1.1)	2.0(1.0)	0.56	0.574	0.001	2.2(1.2)	2.0(1.2)	1.29	0.197	0.005

M, mean; SD, standard deviation; U, Mann–Whitney test; p, significance; ŋ^2^, effect size coefficient; PE, physical education; Days –number of school days; Time – level of physical activity (1 almost none of the time – 5 almost all of the time).

### 3.2 Assessment of school PA (PE lessons, breaks, lunch breaks) before and after the pandemic

**Figure 1:**
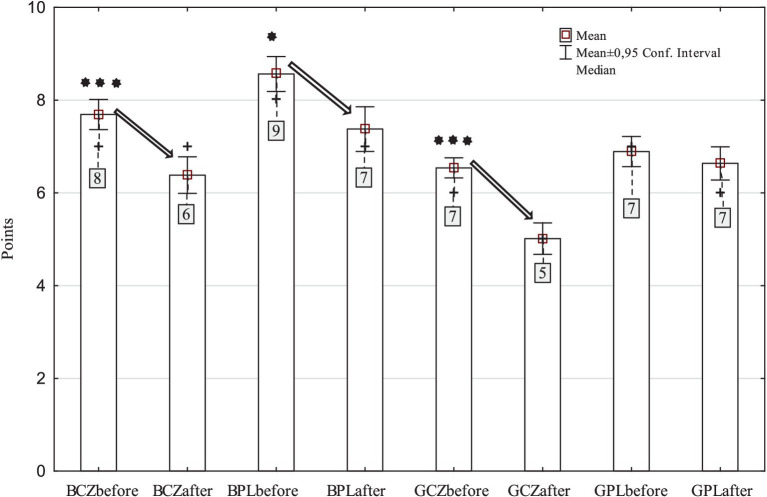
Assessment of school physical activity (physical education, breaks, lunch breaks) of Czech and Polish boys and girls (BCZ–Czech boys, BPL–Polish boys, GCZ–Czech girls, GPL–Polish girls) before and after pandemic (^*^p < 0.05, ^***^p < 0.001).

For the total school PA indicators (PE lessons + breaks and lunch breaks), Czech (*p* < 0.001) and Polish (*p* = 0.018) boys and Czech girls (*p* < 0.001) after pandemic showed significantly less school PA than before pandemic H_(7,2,273)_ = 207.26, *p* < 0.001, *η^2^* = 0.088 (Figure 1). However, the results of this total school PA indicator also included those participants who stated that they did not have PE lessons, breaks or lunch breaks. The missing opportunities before and after pandemic, respectively, were as follows: PE lessons (6.8 and 22.2%), breaks (6.1 and 9.0%) and lunch breaks (22.8 and 24.8%). However, not performing or not participating in these types of school PA is also a significant indicator of the level of school PA.

### 3.3 Assessment of out-of-school PA (to and from school, and before and after school) before and after pandemic

**Figure 2:**
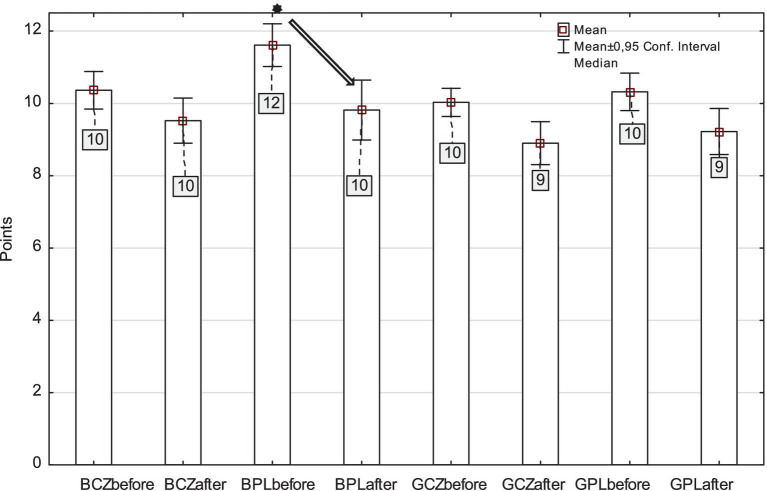
Assessment of out-of-school physical activity (to and from school, before and after school) in Czech and Polish boys and girls (BCZ–Czech boys, BPL–Polish boys, GCZ–Czech girls, GPL–Polish girls) before and after pandemic (^*^p < 0.05).

In out-of- school PA on school days, we found lower PA after pandemic compared to PA before pandemic [H_(7,2,273)_ = 51.54, *p* < 0.001, *η^2^* = 0.020] (Figure 2). Only Polish boys showed a significant group difference in out-of-school PA between before and after school (*p* = 0.020). It became apparent that even out-of-school PA did not replace the post-pandemic decline in school PA.

### 3.4 Assessment of total daily PA (to and from school, school and before and after school) before and after the pandemic

**Figure 3:**
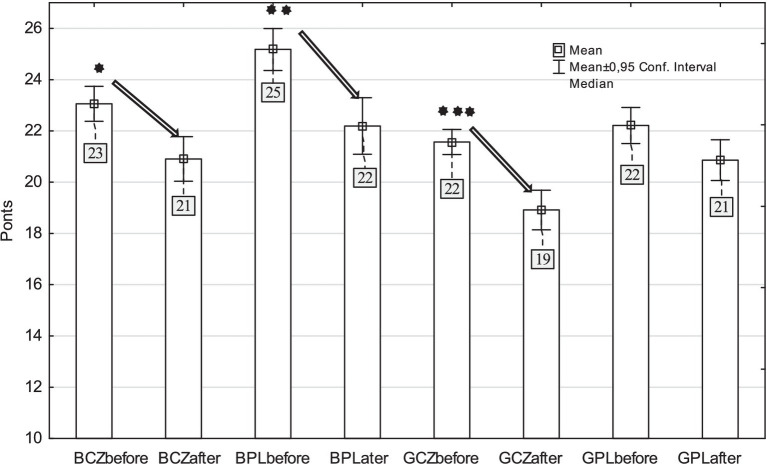
Physical activity assessment on school days (before and after school, to and from school, school physical activity) in Czech and Polish boys and girls (BCZ–Czech boys, BPL–Polish boys, GCZ–Czech girls, GPL–Polish girls) (^*^p < 0.05; ^**^p < 0.01; ^***^p < 0.001).

For the overall point assessment of PA on school days we found a significantly lower level of PA after pandemic than before pandemic H_(7,2,273)_ = 133.77, *p* < 0.001, *η^2^* = 0.056 (Figure 3). This was significant in Czech boys (*p* = 0.017), Polish boys (*p* = 0.001) and Czech girls (*p* < 0.001). Polish boys had higher PA on school days before the pandemic (25 out of 40 points) and Czech girls had the lowest scores after pandemic (19 out of 40 points). The negative effects of the pandemic are also documented by the fact that before the pandemic, a total of 82% of boys and girls said that they enjoyed PA, while only 77% after the pandemic (χ^2^ = 8.92, *p* = 0.003, *r* = 0.059). Similarly, 67% of boys and girls before the pandemic said they enjoyed PE, while only 60% after the pandemic (χ^2^ = 12.09, *p* < 0.001, *r* = 0.073).

### 3.5 Differences in PA in segments of the school days before and after pandemic

**Figure 4:**
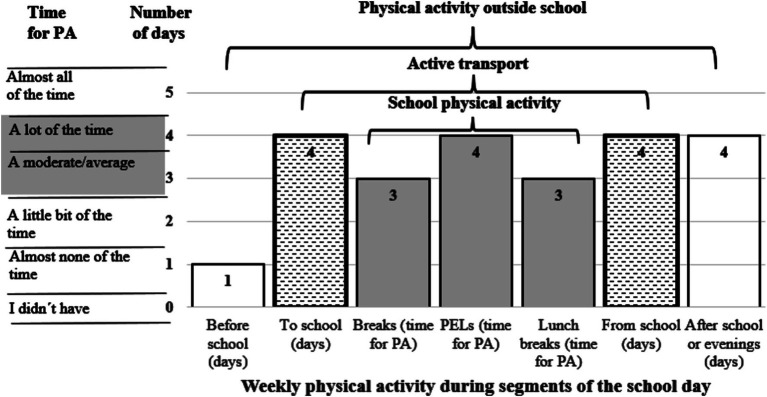
Target recommendations (number of days or time spent) for physical activity in segments of the school days according to the Youth Activity Profile questionnaire.

The recommendations for PA in the segments of the school day are presented in Figure 4. We found a significant difference before and after the pandemic in the achievement of PA recommendations in the segments of the school day for Czech boys (9.7 p.p.), Polish boys (15.1 p.p.) and Czech girls (14.4 p.p.) (Table 3). Similar significant patterns were evident for PA from school for Czech boys (15.2 p.p.), Polish boys (14.2 p.p.), and Czech girls (16.1 p.p.). A significant difference was apparent also in the achievement of the amount of PA in PE lessons among Czech boys (11.1 p.p.) and girls (10.3 p.p.). In Polish girls, the difference was significant in PA during breaks (11.1 p.p.) and PA before school (11.7 p.p.). Differences in the assessment of PE lessons varied significantly between Czech (61.8%) and Polish boys (80.2%) after the pandemic (χ^2^ = 12.76, *p* < 0.001, *r* = 0.073), and also between Czech (43.5%) and Polish girls (66.7%) after pandemic (χ^2^ = 19.04, *p* < 0.001, *r* = 0.091).

**Table 3:** Achievement of physical activity recommendations in segments of the school day Czech and Polish boys and girls before and after pandemic.

Physical activity levels	Czech boys					Polish boys				
	Before *%*	After *%*	χ^2^	*p*	*r*	Before (%)	After (%)	χ^2^	*p*	*r*
School days										
Before school (1 day)	33.5	33.3	< 0.001	0.976	0.001	47.2	40.4	1.80	0.179	0.065
To school (≥ 4 days)	61.6	51.9	5.04	0.025	0.098	71.3	56.2	9.93	0.002	0.152
From school (≥ 4 days)	69.9	54.7	13.02	0.001	0.016	73.8	59.6	9.09	0.003	0.145
After school or evenings (≥ 4 days)	33.5	32.6	0.48	0.827	0.001	36.4	32.9	0.52	0.473	0.035
Time (moderate/average use of the time for PA in PE lessons or breaks)										
During PE lessons	72.9	61.8	6.28	0.012	0.110	76.8	80.2	0.60	0.438	0.037
During breaks	34.3	26.8	3.12	0.078	0.077	49.4	38.2	4.53	0.033	0.102
During lunch breaks	32.4	42.0	4.25	0.039	0.090	51.7	35.7	7.09	0.008	0.128

	Czech girls					Polish girls				
School days										
Before school (1 day)	32.0	29.7	0.42	0.519	0.023	37.2	26.5	6.79	0.009	0.111
To school (≥ 4 days)	65.0	50.6	15.19	<0.001	0.141	66.4	59.1	3.00	0.083	0.074
From school (≥ 4 days)	70.0	53.9	19.76	<0.001	0.160	70.6	66.1	1.25	0.264	0.048
After school or evenings (≥ 4 days)	27.8	29.4	0.21	0.645	0.017	28.5	26.1	0.40	0.525	0.027
Time (moderate/average use of the time for PA in PE lessons or breaks)										
During PE lessons	53.8	43.5	4.88	0.027	0.080	61.7	66.7	1.30	0.253	0.049
During breaks	20.0	20.4	0.02	0.897	0.001	31.4	27.9	0.68	0.409	0.035
During lunch breaks	30.0	28.6	0.13	0.717	0.013	37.7	31.1	1.73	0.188	0.056

M, mean; SD, standard deviation; χ^2^, Pearson’s chi-squared test; p, significance, r, effect size coefficient, PE, physical education; Days – number of school days; Time – level of physical activity (1 almost none of the time – 5 almost all of the time).

In accordance with the results of the study and the ways in which they are presented to school management, teachers and students, we propose the following recommendations for PA in segments of the school day (Figure 4).

The established recommendations for PA in segments of the school day were met in aggregate in both states: Before school (1 day) – 38.6% of boys and 32.0% of girls (*p* = 0.001); To school (≥ 4 days) – 57.3% of boys and 56.5% of girls (*p* = 0.693); From school (≥ 4 days) – 60.0% of boys and 58.9% of girls (*p* = 0.591); After school or evenings (≥ 4 days) – 62. 7% of boys and 50.5% of girls (*p* < 0.001); During PE lessons – 72.8% of boys and 56.9% of girls (*p* < 0.001); During breaks – 37.5% of boys and 24.2% of girls (*p* < 0.001); During lunch breaks – 41.3% of boys and 31.8% of girls (*p* < 0.001). Excluding transport PA to and from school, boys showed significantly more PA than girls.

## 4 Discussion

The study examined youth PA profiles to understand the contributions of different time segments in the school day to advance school PA promotion. We highlighted clear findings with significant differences in PA of Czech and Polish boys and girls before and after the pandemic in transport PA to and from school. It is difficult to determine the main cause of these differences, but disruption of the habit of regular PA seems to be the most likely factor. Other studies have reported a significant decline in overall PA in adolescents in distance education during the pandemic (34, 40), but we expected a prompt return to pre-pandemic levels in transport PA. Especially because an increase in transport PA was also found during the pandemic in adolescents (41) or there was no decrease, for example in Czech and Polish adolescent girls (34). Restoration of pre-pandemic levels of transport PA, or even an increase, should be one of the primary goals of post-pandemic school policy.

Similar to the transport PA, the negative effects of the pandemic were also manifested after the pandemic in the school PA level, especially in PE lessons and breaks, although not for all groups. At the same time, the schools strove to implement a fully valuable standard educational program even in more demanding settings, with the lingering negative effects of the pandemic. Increasing of PA in PE classes and during breaks is particularly relevant for Czech boys and girls, including improving participation in PE lessons. The demand for the participation of all adolescents in PE lessons who are at school (42) is even more relevant in the post-pandemic era. Participation in PE lessons is positively associated with PA and other healthy behaviors (22) and those adolescents who attend PE lessons ≥3 days/week had double the odds of being sufficiently active (20). National, regional and worldwide data highlight the importance of improving participation in PE, particularly for girls and older adolescents (22). According to previous research results, the observed differences between Czech and Polish adolescents are influenced not only by a greater number of PE lessons per week (in most schools in the Czech Republic 2 and in Poland 3 PE lessons), but also by better access to PE and sports in Polish than in Czech schools (35). Polish schools also support students’ involvement in organized PA more (43).

During the pandemic, school PA was lost due to distance education, and this was not replaced by home-based online PE or other ways of supporting PA (34). Difficulties in delivering online PE during the pandemic have also been reported in other studies (44). After the pandemic, online PE should take a completely new position in supporting adolescent PA, supporting the innovative role of physical literacy (45), in supporting less physically active adolescents (46) and in supporting individual PA, with regular use of wearables (34). However, the support and control of quality PE (47), the priority position of PE in the promotion of the “new” role of PA after pandemic (45), as well as the support of the previously requested reform of the professional training of PE teachers, are essential (48).

Also, in total daily PA on school days, we found lower PA after pandemic compared to school days before pandemic. Unfortunately, there was no return of the PA level to the time before the pandemic, as found, for example, in the Alpine regions in adults by Schöttl et al. (49). The results of the study show that less school PA was not replaced by increased outside school PA. In this segment of the school day, we did not find a significant difference between before and after the pandemic. Unfortunately, the YAP questionnaire does not allow to characterize the level of PA in individual school days, but only in summary for school days. Nevertheless, this is an advantage over the IPAQ-A questionnaire, which only allows weekly PA to be characterized (50). PA estimation using YAP questionnaires, supplemented by PA monitoring using wearables, could be beneficial for characterizing PA during school days and for improving the quality of feedback to school management, teachers and students. It is also important because schools will play a crucial role in eliminating the negative effects of the pandemic on adolescent PA and weakening any future restrictions on adolescent PA (51). In this context, schools should also take a more prominent position in the field of public health, through high-quality comprehensive school PA programs responsive to pandemic experience (47).

The differences in level of meeting the recommendations of PA in the segments of the school day before and after the pandemic were presented. While not directly comparable to other studies using the IPAQ-A questionnaire (34), we found similar declines in PA for specific segments of the school day. During the pandemic, there was a decrease in the number of days with active transport and a decrease in PA in schools and leisure activities.

Another study indicated that the recommendation for PA before school (at least 2,000 steps) was met by 29% of boys and 38% of girls, and the recommendation of at least 10 min of moderate to vigorous PA ≥ 3 METs was met by 37% of boys and 27% of girls (25). Adolescents’ transport PA to high schools is more complex to research than children’s transport PA to schools because of the greater diversity of settings, which is reflected in both the research focus and the smaller number of studies and interventions (52). The number of school days with transport PA to and from schools per week is an important indicator for management and teachers at schools in a way to support active transport to school with the arrangement of suitable and safe facilities for this type of PA.

The significantly worse assessment of the time spent in PA in PE lessons after pandemic by Czech boys and girls requires attention as Polish boys and girls rated PA in PE lessons after pandemic better, although not significantly. It is very difficult to determine the causes of these differences between the education system in both countries. This could be due to already mentioned differences in the number of PE lessons per week and the status of sport in schools, the predominance of single-subject PE teachers in Poland (in the Czech Republic, PE teachers are mostly educated in two disciplines) or the previously reported better evaluation of PE lessons by Polish boys and girls (23).

The aggregate adherence to PA recommendations in each segment of the school day, except for transport PA to and from school, confirms greater adherence to PA recommendations by boys than girls. Similarly, greater adherence of boys to PA recommendations was found in Norwegian adolescents by Grasaas and Sandbakk (53), therefore underlining the importance of promoting adherence to PA recommendations, especially among girls. The emphasis on promoting girls’ PA is also significant because girls show less motivation to PA than boys (54).

Simplified feedback on PA in segments of the school day and total daily PA on school days was positively received by schools. The school management’s request was primarily focused on how students meet the recommendations for PA and what will be the future most appropriate focus in supporting PA and a healthy lifestyle for students. European studies show a clear demand for policy makers to immediately address the negative impact of the pandemic on PA in children and adolescents, in line with an evidence-based public health strategy (55).

However, the positive evaluation of the use of the YAP questionnaire in schools in this study should be taken very cautiously, as it is based only on the statement of the school management and teachers that they want to continue the similar assessment of students’ PA continuously.

We consider the obtained results regarding the level of school PA according to the YAP questionnaire, even in the simplified form of the level of frequency or time spent in PA for schools, to be stimulating and sufficient for choosing a strategy to increase PA on school days.

### 4.1 Limitations

The limitation of the study is that it was not possible to obtain a representative set of schools for the research. A significant limitation was that school management did not allow, due to more serious educational tasks, to reach the same students involved in the research before the pandemic. Even so, the almost two-year gap, changes in schools and the influence of other presenters on the lifestyle of the probands would be very limiting.

Differences in student participation in PE lessons, breaks or lunch breaks are also a limitation of the study. Non-participation of students in PE lessons was not possible to be compared with the actual number of registered and implemented PE lessons at schools.

The results of this research call for intensive monitoring of long-term trends in PA in high school students in the post-pandemic period. It also calls for analysis of how schools will change their approach to promoting online PE and preparing students for PA in distance education regarding potential restrictions in the future. Equally important are the suggestions for changes in the professional training of PE teachers.

## 5 Conclusion

The pandemic disrupted the habit of regular PA during school days, which was particularly reflected in lower levels of transport PA to and from school and in school PA in both Czech and Polish high schools. These findings underline the importance of student participation in PE classes, promoting regular organized PA in the school’s extra-curricular program, promoting popular forms of transport PA to and from school, and enhancing cooperation between schools and organizations ensuring equal access to leisure-time PA for all students. Promoting achievement of the recommendations in the segments of the school day and in comprehensive school PA programs should be an important part of school health and education policy and public health promotion for adolescents. Providing online PE for students should be mandatory in schools and inclusion of PA referrals in comprehensive school PA programs controlled. Opportunities to provide wearables for individual PA monitoring by students from school resources should be improved. Future research on adolescent school PA should focus on the use of YAP in combination with monitoring PA, sedentary behavior and sleep using wearables. Importantly, it should include qualitative research on the school lifestyle of students, teachers and school administrators.

## Data Availability

The raw data supporting the conclusions of this article will be made available by the authors, without undue reservation.

## Footnotes

^1^ www.indares.com
